# Dynamic interactions between physical activity, exercise adherence, and adverse psychological states in Chinese older adults: a cross-lagged network analysis

**DOI:** 10.3389/fpubh.2026.1744350

**Published:** 2026-03-26

**Authors:** Luyao Xiang, Hao Gou, Jing Yang, Chang Hu, Dong Zhu, Bo Xu

**Affiliations:** 1 Zhuhai Campus, Zunyi Medical University, Zhuhai, China; 2 Hunan University of Medicine, Huaihua, China; 3 College of Physical Education, Qiannan Normal University for Nationalities, Duyun, China; 4 Mianyang Normal University, Mianyang, China; 5 College of Physical Education, Jiangxi Normal University, Nanchang, China

**Keywords:** adverse psychological states, cross-lagged network analysis, dynamic relationship, older adult population in China, exercise adherence, physical activity

## Abstract

**Background:**

With the accelerating process of population ageing in China, negative emotional problems in older people have become increasingly prominent. Exploring the dynamic relationship between these emotions and physical activity, as well as exercise adherence, is of great significance for promoting the physical and mental health of older people.

**Methods:**

This study used a longitudinal design to conduct two follow-up surveys of 876 older adult individuals in China. A cross-lagged network analysis method was employed to examine the interactions between physical activity, exercise adherence, and adverse psychological states indicators (depression, anxiety, stress, loneliness, and sadness/anger rumination).

**Results:**

Cross-sectional network analysis showed that physical activity and exercise adherence maintained a stable relationship, and the network structure tended to become more concentrated over time. The cross-lagged analysis found that exercise adherence not only showed good temporal stability (*β* = 0.233) but also significantly predicted subsequent levels of physical activity (*β* = 0.131) and had a negative predictive effect on stress (*β* = −0.129), anxiety (*β* = −0.081), and loneliness (*β* = −0.079). At the same time, adverse psychological states formed a mutually reinforcing network structure, with stress and anxiety having higher centrality in the system.

**Conclusion:**

Exercise adherence plays a key role in the physical and mental health of the older adult in China, serving as an important bridge between physical activity and psychological wellbeing. The findings provide an important basis for developing targeted health promotion programs for older people, suggesting that emphasis should be placed on the formation and maintenance of exercise habits, with phased and multi-level intervention strategies.

## 1 Introduction

As China’s ageing population deepens, the increasing proportion of older people has brought about multiple social and public health challenges (1, 2). Against this backdrop, mental health issues within the older adult population have become increasingly prominent, particularly the widespread presence of adverse psychological states, which deserve focused attention (3–5). The adverse psychological states addressed in this study primarily include dimensions such as depression, anxiety, stress, and loneliness (6). These emotional states not only severely impair an individual’s subjective wellbeing but may also lead to physiological decline, diminished social role functions, and an overall decrease in quality of life, thereby creating a mutually reinforcing vicious cycle (7, 8). Therefore, exploring the factors and mechanisms underlying the development of adverse psychological states in older people has become a crucial and urgent research direction in the field of older adult health.

When exploring intervention pathways for adverse psychological states, physical activity is considered a key protective factor (9, 10). At the theoretical level, physical activity may be associated with lower emotional distress through neurobiological mechanisms and psychosocial pathways, such as promoting the release of endogenous neurotransmitters and increasing opportunities for social participation (11). Empirical studies also commonly indicate that regular physical activity is associated with lower levels of adverse psychological states (12, 13). However, the relationship between the two may be bidirectional: adverse psychological states may also significantly hinder an individual’s participation in physical activity by weakening behavioural motivation and energy levels (14, 15). This complex interplay forms the basic theoretical framework for this study’s exploration of the relationship between physical activity and adverse psychological states.

The health benefits of physical activity largely depend on its regular, long-term nature, which is a core element of exercise adherence (16, 17). Exercise adherence not only reflects the stability of health behaviours but may also buffer adverse psychological states by establishing positive feedback mechanisms and strengthening social support networks (18). Theoretical models, such as self-determination theory, suggest that high levels of exercise adherence help satisfy an individual’s basic psychological needs, thereby enhancing psychological resilience and resisting adverse psychological states (19). Empirical observations also support this, showing that older adult individuals who maintain long-term exercise habits typically perform better in emotional regulation (20, 21). On the other hand, strong adverse psychological states may directly weaken an individual’s willingness and ability to adhere to exercise, leading to interruptions or cessation of exercise behaviours (22). Therefore, a dynamic, mutually constraining relationship is also believed to exist between exercise adherence and adverse psychological states, underscoring the need for in-depth analysis of both.

Drawing on self-determination theory (19), social cognitive theory (23), conservation of resources theory (24), and emotional network theory (25), we propose an integrative *a priori* model in which exercise adherence reflects a motivational–self-regulatory capacity (supported by need satisfaction and self-efficacy) that helps sustain physical activity over time; in turn, sustained adherence and activity are expected to be linked with lower adverse psychological states via physiological and psychosocial pathways, whereas higher distress may deplete motivational and energetic resources, undermining subsequent adherence and activity (26). Consistent with emotional network theory, we further expect adverse psychological state indicators to form an interconnected symptom network, with core nodes (e.g., stress and anxiety) potentially reinforcing other symptoms (27). Based on this framework, we hypothesized that: (H1) physical activity and exercise adherence would show a stable positive association at both T1 and T2; (H2) adverse psychological state indicators would exhibit a coherent cluster, particularly the DEP–ANX–STR triad; (H3) exercise adherence would prospectively predict higher subsequent physical activity and lower subsequent adverse psychological states, whereas stress and loneliness would prospectively predict lower subsequent physical activity and/or exercise adherence; and (H4) within the distress subsystem, stress and anxiety would display reciprocal prospective associations and would predict subsequent depressive symptoms.

However, traditional cross-sectional designs and variable-centred analytical approaches are limited in their ability to capture the complex, time-varying interplay among physical activity, exercise adherence, and these adverse psychological state indicators (28, 29). They are unable to reveal the temporal directionality and dynamic evolution of interactions between variables. Network analysis provides a novel paradigm by treating psychological constructs as interconnected variable systems and visually presenting their relationships through network structures (30, 31). Among these, cross-lagged network analysis integrates the dual advantages of longitudinal panel data and network models (32, 33). It not only portrays the covariance structure of variables at multiple time points but also effectively infers the directional predictive relationships between variables, identifies key bridging variables that drive changes within the system, and explains the dynamic evolutionary characteristics of relationships over time (34, 35). The application of this method is expected to systematically reveal the underlying mechanisms and key pathways of the mutual influences between physical activity, exercise adherence, and adverse psychological states, thereby providing solid empirical evidence for targeted, phased psychological and behavioural intervention strategies for older adult individuals in China.

## 2 Methods

### 2.1 Participants and procedures

This study is a prospective longitudinal research, employing purposive sampling to recruit older adult individuals who meet the inclusion criteria from communities, nursing homes, and senior universities in Zhuhai City, Guangdong Province; Guiyang City and Duyun City in Guizhou Province; and Nanchang City in Jiangxi Province. These recruitment sites are predominantly urban, and the present sample therefore primarily represents community-dwelling older adults in the selected southern provinces. The baseline survey (Time 1, T1) was conducted from June to August 2024, yielding 989 valid questionnaires. The follow-up survey (Time 2, T2) was completed from June to August 2025, with 876 older adult individuals ultimately participating in both rounds of assessment (T1 and T2) and being included in the analysis.

To address missing data, the study employed multiple imputation via chained equations using the mice package in R (36). To account for potential bias due to sample attrition, we further compared baseline characteristics between participants who completed both rounds of the survey and those who participated only in the baseline survey (37). Independent sample t-tests showed that there were no significant differences between the two groups in terms of gender (*t* = −0.091, *p* > 0.05), education level (*t* = 1.722, *p* > 0.05), marital status (*t* = −1.270, *p* > 0.05), living situation (*t* = 2.245, *p* > 0.05), baseline physical activity level (*t* = 0.688, *p* > 0.05), exercise adherence (*t* = −0.878, *p* > 0.05), depression-anxiety-stress (*t* = −0.870, *p* > 0.05), loneliness (*t* = 0.838, *p* > 0.05), or sadness and anger rumination (*t* = 0.321, *p* > 0.05). These results suggest that sample attrition did not introduce systematic bias. These results suggest that completers and dropouts were broadly comparable on the observed baseline variables; however, attrition due to unmeasured factors (e.g., health decline, functional limitations, motivation) cannot be fully ruled out.

Among the 876 older adult individuals included in the analysis (see Table 1), 345 were male (39.4%), and 531 were female (60.6%). The age distribution was as follows: 530 individuals (60.5%) were aged 60–69 years, 254 individuals (29.0%) were aged 70–79 years, and 92 individuals (10.5%) were aged 80 years and above. In terms of education level, 364 individuals (41.6%) had education up to or below primary school, 265 individuals (30.3%) had completed middle school, 166 individuals (18.9%) had completed high school, and 81 individuals (9.2%) had a college education or higher. Regarding marital status, 617 individuals (70.4%) were married, and 259 individuals (29.6%) were unmarried. As for living arrangements, 383 individuals (43.7%) lived alone, while 493 individuals (56.3%) lived with their children.

**Table 1:** Basic characteristics of the sample.

Variable	Sort	Frequency	Scale
Sex	Male	345	39.4%
	Female	531	60.6%
Education	Primary school or below	364	41.6%
	Middle school	265	30.3%
	High school	166	18.9%
	College or above	81	9.2%
Age	60–69 years	530	60.5%
	70–79 years	254	29.0%
	80 years and above	92	10.5%
Marital status	With spouse	617	70.4%
	Without spouse	259	29.6%
Living situation	Living alone	383	43.7%
	Living with children	493	56.3%

### 2.2 Measuring tools

#### 2.2.1 Physical Activity Rating Scale (PARS-3)

Physical activity in older people was assessed using the Physical Activity Rating Scale (PARS-3) developed by Liang (38), which has demonstrated good reliability and validity in older adult populations in China (39). This scale evaluates physical activity across three dimensions: exercise intensity, frequency, and duration, using a five-level scoring system. The Cronbach’s alpha coefficients for the two measurements (T1, T2) were 0.858 and 0.857, respectively, indicating good internal consistency reliability of the scale.

#### 2.2.2 Exercise Aherence Scale (EAS)

In this study, exercise adherence was assessed using the Exercise Adherence Scale (EAS) developed by Wang Shen and colleagues (40). The scale consists of 14 items and uses a 5-point scale (1 = “Strongly disagree,” 5 = “Strongly agree”), with higher scores indicating greater exercise adherence. The scale includes three dimensions: behavioural habits, effort investment, and emotional experience. The internal consistency coefficients for each dimension are shown in Supplementary Table S1. In this study, the Cronbach’s *α* coefficients for the total scale were 0.889 at T1 and 0.898 at T2. Confirmatory factor analysis indicated good structural validity of the scale, with specific fit indices provided in Supplementary Table S1.

#### 2.2.3 Depression Anxiety Stress Scales – 21 Items (DASS-21)

Based on the Depression Anxiety Stress Scales – 21 Items (DASS-21) developed by psychologist Lovibond and Lovibond (41), this study systematically adapted a Chinese version of the shortened scale through cultural and linguistic adjustments. The revision process strictly followed cross-cultural scale adaptation guidelines, completing key steps including forward translation, back translation, expert committee review, and a pre-test (42). Bilingual psychology researchers conducted the initial translation, and the back translation was performed by English-speaking researchers who had not been exposed to the original scale, thereby ensuring conceptual equivalence. Subsequently, a committee of clinical psychologists and experts in geriatric linguistics reviewed each item, adjusting the phrasing to align with the cultural and linguistic habits of older adult individuals in China. During pre-test cognitive interviews, any potentially ambiguous wording was further refined, resulting in a Chinese version of the DASS-21 with good face and content validity. This scale consists of 21 items, covering three dimensions: depression, anxiety, and stress, using a 4-point scoring system (0 = “Never” to 3 = “Always”), with higher total scores indicating more severe adverse psychological states.

To examine its psychometric properties, this study conducted a systematic analysis of longitudinal data collected at two time points. The results of confirmatory factor analysis indicated that the scale had good structural validity at both time points (specific fit indices are shown in Supplementary Table S1). In terms of reliability, the Cronbach’s *α* coefficients for the total scale at T1 and T2 were 0.915 and 0.916, respectively. The internal consistency coefficients for each dimension are detailed in Supplementary Table S2, indicating that the scale demonstrates excellent measurement stability and reliability within the older adult population in China.

#### 2.2.4 University of California, Los Angeles (UCLA) Loneliness Scale – short form (ULS-6)

Loneliness was measured using the short version of the University of California, Los Angeles (UCLA) Loneliness Scale (ULS-6) (43). This scale consists of 6 items and uses a 4-point scale (1 = “Never” to 4 = “Always”), with higher total scores indicating greater loneliness. In this study, the process of adapting and culturally adjusting the scale followed the same steps outlined earlier, ensuring its cultural applicability to the older adult population in China. Reliability analysis showed Cronbach’s *α* coefficients of 0.880 at T1 and 0.877 at T2, indicating excellent internal consistency. The results of confirmatory factor analysis demonstrated good structural validity of the scale (specific fit indices are provided in Supplementary Table S1).

#### 2.2.5 Sadness and Anger Rumination Questionnaire (SARQ)

This study used the Sadness and Anger Rumination Questionnaire (SARQ) to measure rumination in the subjects (44). The process of adapting and culturally adjusting the questionnaire followed the same steps outlined previously. The final version consisted of 22 items, divided into two dimensions: sadness rumination and anger rumination. It uses a 5-point scoring system (1 = “Never” to 5 = “Always”), with dimension scores calculated by averaging item scores, where higher scores indicate higher levels of rumination in the corresponding category. In this study, the internal consistency coefficients for each dimension are shown in Supplementary Table S2. The Cronbach’s *α* coefficient for the total scale was 0.918 at T1 and 0.930 at T2. Confirmatory factor analysis indicated good structural validity of the scale, with specific fit indices provided in Supplementary Table S1.

### 2.3 Data analysis

#### 2.3.1 Preliminary analysis

Descriptive statistics and correlation analysis were performed for each variable at both T1 and T2. Independent-samples *t*-tests were then conducted to compare differences in core variables between older adult individuals who dropped out at T2 and those who continued participating, to assess potential systematic biases introduced by sample attrition. Based on these results, one-way analysis of variance and independent sample t-tests were used to identify variables with significant differences in demographic characteristics, which were then included as covariates in the subsequent network analysis model. Specifically, after multiple imputation, we adjusted for these covariates by residualizing each core variable at each time point (dummy-coding categorical covariates) and used the standardized residuals to estimate both the cross-sectional and cross-lagged networks; thus, reported edges represent conditional associations after accounting for these demographics (45). Additionally, Harman’s single-factor test was performed on both T1 and T2 data to assess common method bias. However, because Harman’s single-factor test has limited sensitivity, it was used only as an initial screening tool; thus, residual common method variance cannot be fully excluded in the present dataset (46). All of these analyses were conducted using SPSS 27.0.

#### 2.3.2 Network analysis

The network analysis was performed in R 4.3.2 and included both cross-sectional and cross-lagged panel network analyses (47). First, cross-sectional networks were constructed separately from T1 and T2 data, using a mixed graphical model for estimation and the EBICglasso algorithm for regularisation to simplify the network structure. The hyperparameter *γ* for the Extended Bayesian Information Criterion (EBIC) was set to 0.5 (48). In terms of node centrality assessment, the expected influence was used as a measure of node impact, as this metric shows good stability across networks with mixed polarity (49). All network models were estimated using covariate-adjusted, residualized variables (gender, age, education, marital status, and living situation), so edges can be interpreted as associations conditional on these covariates rather than as simple zero-order relationships.

Furthermore, the study introduced a cross-lagged panel network model to examine the temporal relationships among variables (50). This model integrates autoregressive and cross-lagged paths, allowing evaluation of how other variables predict a given variable while controlling for the variable’s own previous levels. The specific modelling process was implemented using the R package glmnet, and visualization was completed with the qgraph package. To identify key driving and sensitive nodes in the network, the study further examined centrality indices that reflect the extent to which a node is influenced by other variables and the extent to which it predicts other variables (51).

Finally, the Bootstrap sampling method was used with 1,000 iterations to calculate the 95% confidence intervals for the edge weights to assess the estimation accuracy and examine the stability of the centrality indices. The stability coefficient (CS) was used as a measure of stability, with CS > 0.25 considered acceptable and CS > 0.5 indicating good stability (52).

## 3 Results

### 3.1 Common variance test

In this study, Harman’s single-factor test was used to analyze common method bias. The results showed that, across both time points, 10 factors had eigenvalues greater than 1 (46). The first factor accounted for 19.25% and 18.41% of the variance at T1 and T2, respectively, both of which were below the 40% threshold. This indicates that there is no significant common method bias in the data of this study.

### 3.2 Descriptive analysis

To provide a comprehensive description of the sample characteristics, Supplementary Table S3 presents the descriptive and correlation statistics for each variable at both time points. Based on these results, one-way analysis of variance and independent-samples t-tests were conducted to examine the impact of demographic variables on the core study variables. The findings revealed that gender, age, education level, marital status, and living situation significantly affected the scores of various variables. Therefore, in the subsequent network analysis, these demographic variables were included as covariates in the model to control for their effects. In practice, these covariates were not included as additional nodes; instead, we regressed each main variable on the covariates at T1 and T2 and conducted network estimation on the resulting covariate-adjusted (standardized) residuals.

### 3.3 Cross-sectional network analysis

**Figure 1:**
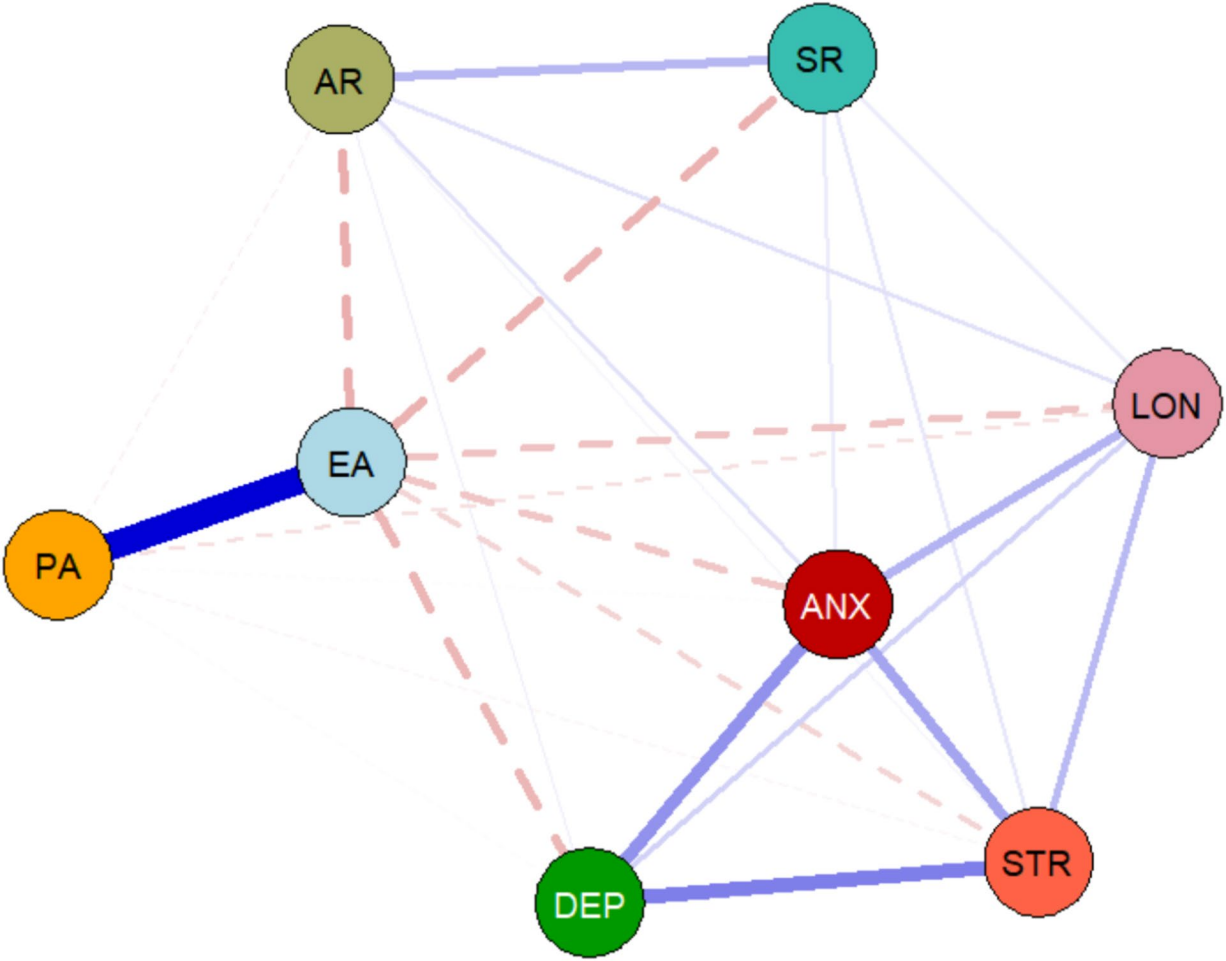
Cross-sectional network structure of physical activity, exercise adherence, and adverse psychological states at T1. Nodes represent the variables, and edges represent the partial correlations between the variables. Green edges indicate positive correlations, while red edges indicate negative correlations. The thickness of the edges is proportional to the strength of the correlation. Variables: Physical activity (PA), Exercise adherence (EA), Depression (DEP), Anxiety (ANX), Stress (STR), Loneliness (LON), Anger rumination (AR), Sadness rumination (SR).

**Figure 2:**
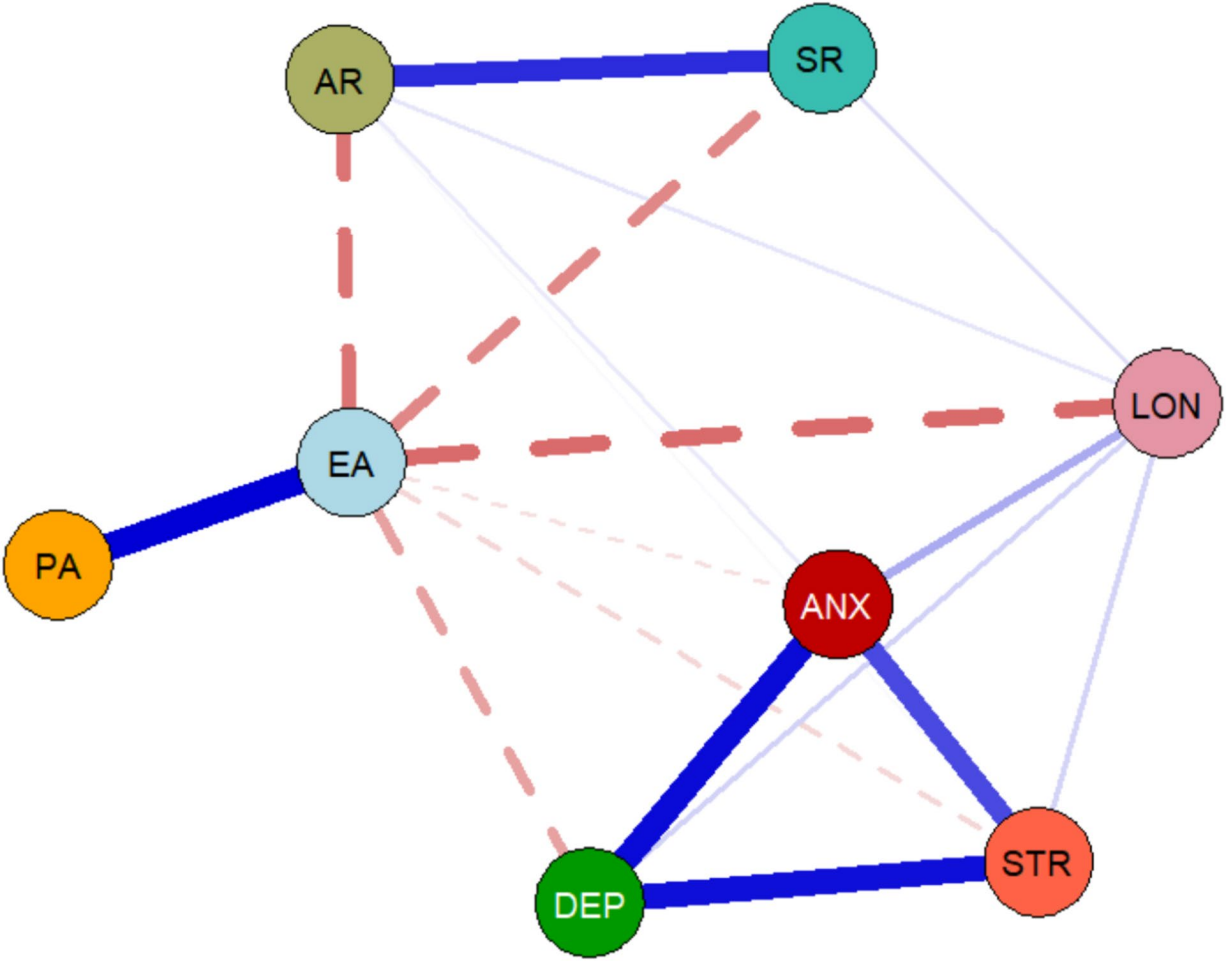
Cross-sectional network structure of physical activity, exercise adherence, and adverse psychological states at T2. The network structure at T2 is sparser and more concentrated than at T1.

This study first analyzed the cross-sectional network structures at both time points. At T1, the network contained 26 non-zero edges, with a network density of 0.9286, indicating that 92.86% of all possible variable connections were present and suggesting extensive and complex interactions between the variables. As shown in Figure 1, the most prominent relationship at this time point was the moderate positive correlation between physical activity and exercise adherence (*r* = 0.447), suggesting that individuals with higher levels of physical activity also tended to have higher exercise adherence. Additionally, exercise adherence showed negative correlations with anger rumination (*r* = −0.143) and sadness rumination (*r* = −0.136), suggesting that regular exercise may be associated with reduced rumination of these two types. In addition to these behaviour-related links, the psychological symptom nodes were also interconnected at T1: depression (DEP), anxiety (ANX), and stress (STR) showed concurrent positive connections with each other, indicating a coherent distress cluster already present at baseline, although these links appeared less pronounced than at T2. At T2 (Figure 2), the network structure changed significantly. The number of non-zero edges decreased to 18, and the network density dropped to 0.6429 (64.29% of edges were non-zero), a 28.57% decrease from T1. This suggests that the associations between variables became sparser and more concentrated over time. The positive correlation between physical activity and exercise adherence remained one of the strongest connections, but its strength weakened (*r* = 0.269). At the same time, the negative correlation between exercise adherence and loneliness became more pronounced (*r* = −0.156), emerging as a relatively strong association at T2, suggesting that, over time, the relationship between exercise adherence and reduced loneliness may have become stronger.

Additionally, the negative correlation between exercise adherence and anger rumination persisted (*r* = −0.146). Moreover, the DEP–ANX–STR cluster was more salient at T2, with stronger mutual connections among these core distress symptoms, suggesting tighter coupling within the psychological subsystem over time. A comprehensive review of cross-sectional network results from both time points shows sustained but dynamically changing associations among physical activity, exercise adherence, and indicators of adverse psychological states (particularly rumination and loneliness). Moreover, the network’s overall connectivity decreased over time. Detailed values of the edges in all cross-sectional networks are presented in Supplementary Tables S4, S5.

**Figure 3:**
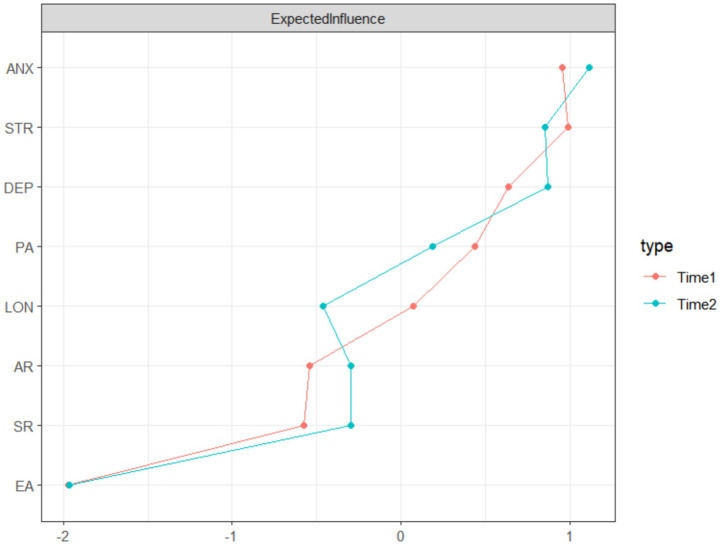
Expected influence (EI) centrality indices of cross-sectional networks at T1 and T2. This figure shows the relative influence of each variable in the network at both time points. The higher the EI value, the more central the node is in terms of connectivity and influence within the network.

The analysis results show that at T1, the variable “stress” (STR) has the highest centrality (EI = 0.989), indicating that it is the most active and influential node in the network, suggesting that it was among the most interconnected nodes at baseline (in terms of expected influence). By T2, the network’s core shifted, with “anxiety” (ANX) becoming the node with the highest centrality (EI = 1.112), and its influence increased relative to the stress node at T1. This dynamic change in centrality suggests that, within the Chinese older adult population, the relative importance of different adverse psychological states in the psychological-behavioural network evolves. Initially, stress may be the central focus, and anxiety may represent a potentially salient node for further investigation and intervention planning. Specific centrality indices for all nodes at both time points are detailed in Supplementary Table S6. Notably, because some centrality indices (e.g., closeness) showed only borderline stability at T1, interpretations of node “importance” should remain cautious and be corroborated in future studies (see Figure 3).

This study used the Bootstrap sampling method to conduct stability testing of the cross-sectional networks, evaluating the reliability of the network structure through the correlation stability coefficient (CS coefficient). At T1, the stability of key indicators, such as edge weights (CS = 0.672) and expected influence (CS = 0.672), was good, indicating that the estimates of network connectivity strength and node centrality were reliable. In contrast, the stability of indicators such as mediator centrality (CS = 0.595) and closeness centrality (CS = 0.517) was relatively lower. At T2, network stability was even more favourable, with the CS coefficient for edge weights reaching 0.75 and the expected influence indicator maintaining a high level of stability (CS = 0.672). The bridge expected influence indicators at both time points showed good stability, with the CS coefficients being 0.672 at both T1 and T2. Overall, the stability of the main indicators in both cross-sectional networks reached an acceptable level, providing the necessary reliability for subsequent network comparisons and result interpretations. The Bootstrap confidence intervals for edge weights and the results of centrality difference tests are provided in the Supplementary materials.

### 3.4 Cross-lagged network analysis

**Figure 4:**
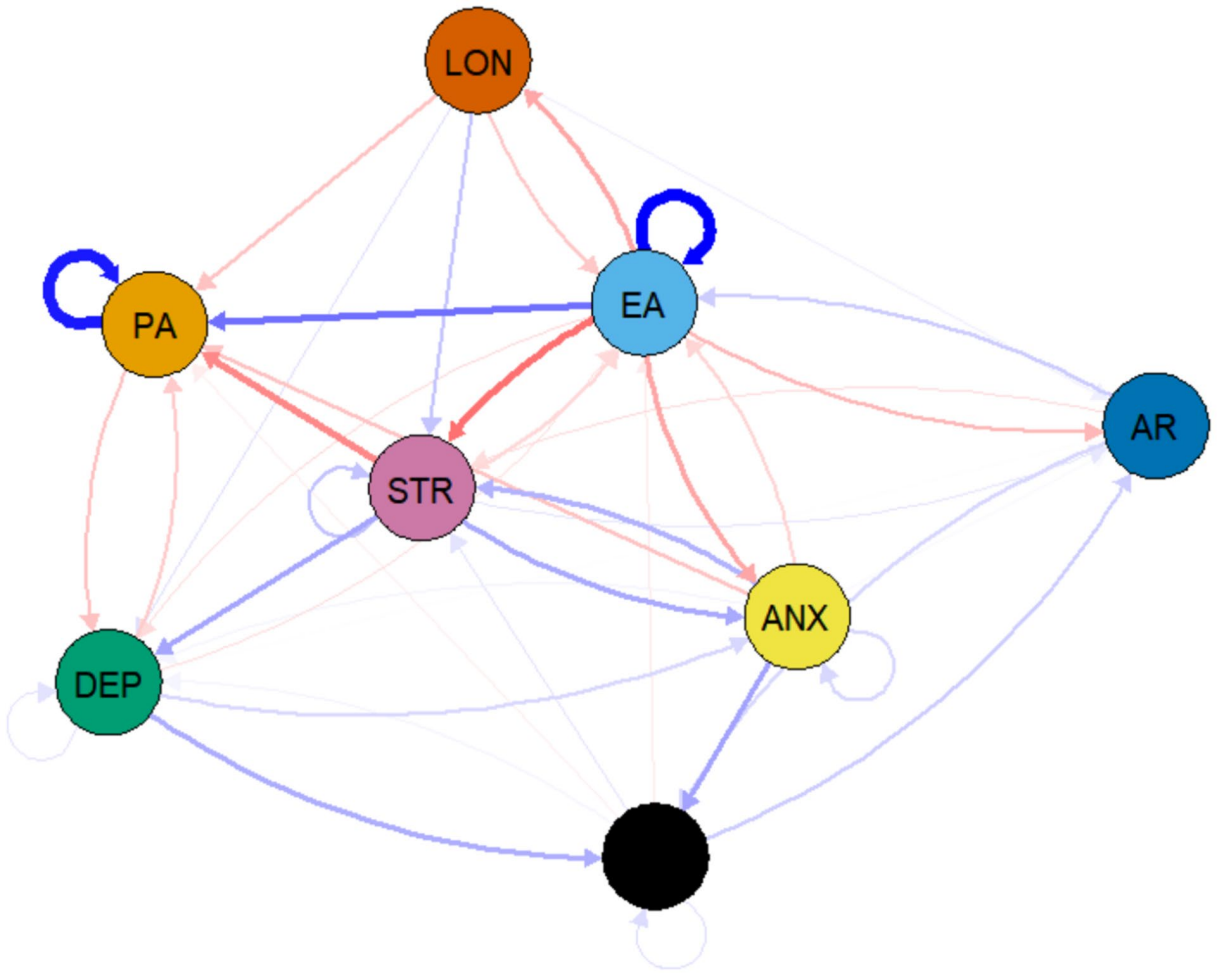
Cross-lagged network of physical activity, exercise adherence, and adverse psychological states. Solid arrows represent positive predictive paths, while dashed arrows represent negative predictive paths. The thickness of the arrows is proportional to the size of the regression coefficients (*β*). The figure illustrates the temporal relationships among variables.

This study further explored the longitudinal predictive relationships between variables using cross-lagged network analysis. The network contained 42 non-zero edges, revealing the dynamic interplay between physical activity, exercise adherence, and adverse psychological states. The analysis results showed that autoregressive effects were generally present between the variables, with exercise adherence (*β* = 0.233) and physical activity (*β* = 0.210) exhibiting strong temporal stability. Among the cross-lagged effects, the most influential predictive path was the positive association between exercise adherence and subsequent physical activity (*β* = 0.131), indicating that individuals who maintain exercise adherence tend to sustain higher levels of physical activity in the future. Additionally, exercise adherence showed significant protective effects on various adverse psychological states, particularly negative associations with stress (*β* = −0.129), anxiety (*β* = −0.081), and loneliness (*β* = −0.079). On the other hand, adverse psychological states also had noticeable inhibitory effects on health behaviours. Stress significantly negatively predicted physical activity (*β* = −0.108), while loneliness not only negatively predicted physical activity (*β* = −0.055) but also negatively impacted exercise adherence (*β* = −0.050). Within the internal interactions of adverse psychological states, stress had a significant positive predictive effect on both depression (*β* = 0.081) and anxiety (*β* = 0.078), while anxiety also positively predicted stress (*β* = 0.070), forming a mutually reinforcing vicious cycle. The specific coefficients for these core predictive paths are provided in Supplementary Table S7 (see Figure 4).

**Figure 5:**
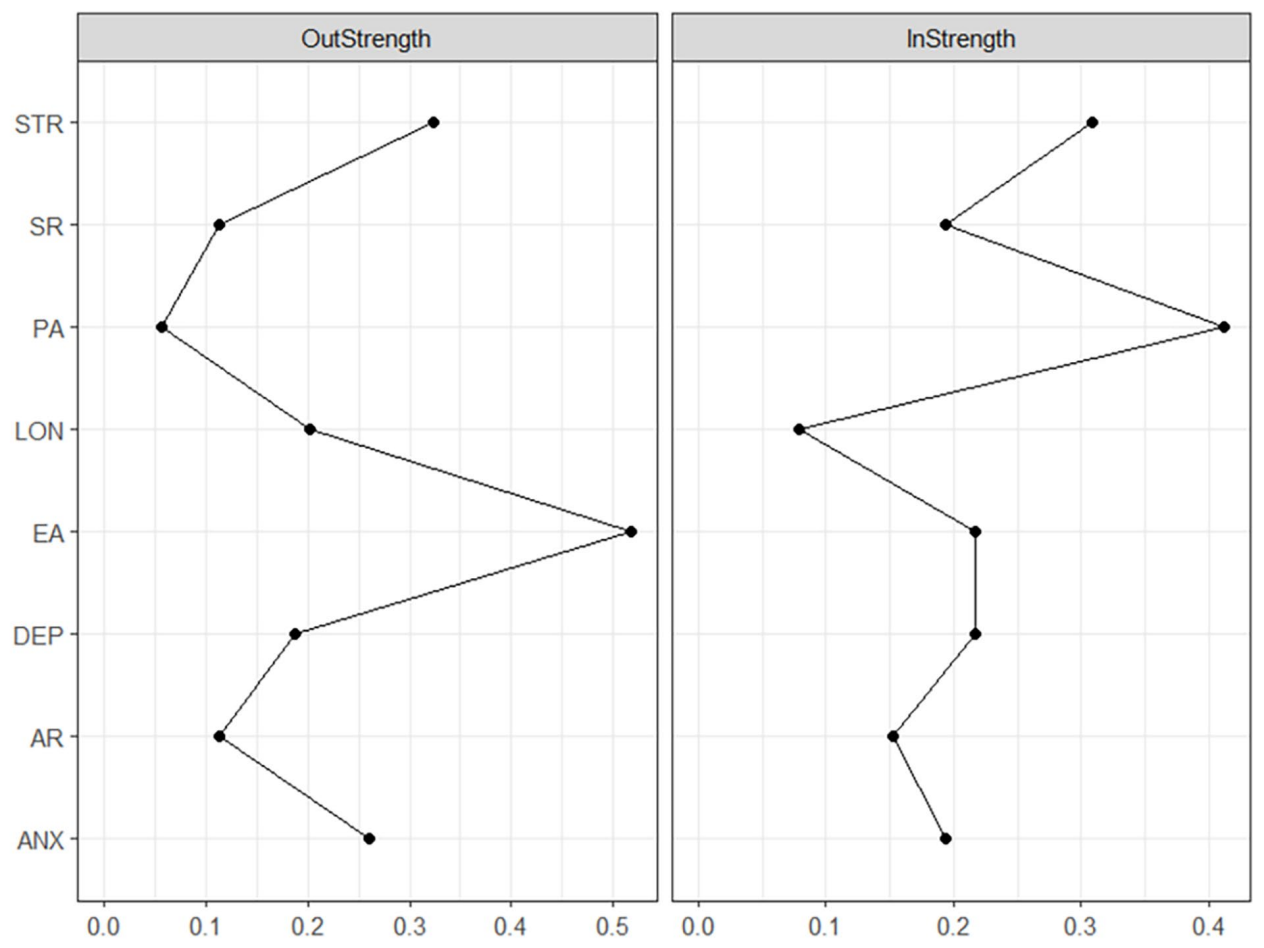
Inward and outward expected influence centrality in the cross-lagged network. Outward expected influence (OutStrength) measures a node’s ability to predict other nodes, while inward expected influence (InStrength) measures the extent to which other nodes predict a node.

The centrality analysis results for the cross-lagged network, shown in Figure 5, reveal that among all observed variables, exercise adherence (EA) exhibits the strongest outward expected influence (OEI = 0.518), indicating that it has the greatest predictive influence on other variables within the system. Meanwhile, physical activity (PA) shows the strongest expected inward influence (IEI = 0.412), suggesting that this variable is most susceptible to influence from other variables in the system. This centrality pattern indicates that exercise adherence primarily drives the system, actively influencing other variables, while physical activity is more reactive, influenced by changes in other factors within the system. The centrality indices for all nodes are provided in Supplementary Table S8.

The stability test results for the cross-lagged network analysis showed that the correlation stability coefficient for edge weights was 0.438, which meets the acceptable standard, indicating that the predictive relationships between variables are fundamentally reliable. At the same time, the stability coefficient for outward centrality was 0.361, meeting the stability criterion and suggesting that nodes measured as predictors are credible. These indicators provide necessary reliability support for the key structural features of the cross-lagged network. The detailed Bootstrap confidence intervals are provided in the Supplementary materials.

## 4 Discussion

### 4.1 Dynamic features of cross-sectional network structure

This study revealed the dynamic features of the relationships among physical activity, exercise adherence, and adverse psychological states using cross-sectional network analysis at two time points among older adult individuals in China. One notable finding is that, from T1 to T2, the network density decreased significantly by 28.57%, indicating that the associations between variables became more sparse and concentrated overall. Conceptually, this sparsification may indicate an adaptive reorganization of the psychological-behavioural system, in which only the most robust and stable connections persist while weaker or context-dependent links fade (53). From a complex-systems and network perspective, a decrease in density can also reflect increased modularity, meaning that behaviour-related and distress-related nodes become more differentiated over time (54). One plausible explanation is that, as older adults consolidate exercise routines and coping strategies across the year, physical activity and exercise adherence may become less contingent on short-term fluctuations in distress, whereas distress indicators may be increasingly shaped by broader influences (e.g., chronic health changes or social circumstances) that are not explicitly modeled in the present network; consequently, fewer stable cross-domain edges remain detectable at T2. Another possibility is that ageing-related experiences diversify over time, increasing between-person heterogeneity; under regularized network estimation, this can lead to fewer edges surviving at T2 because associations are less consistent across individuals. Importantly, lower density should not be interpreted as inherently better or worse; rather, it suggests a structural reconfiguration that helps prioritize the stable, clinically meaningful links that persist over time when designing interventions (55).

At T1, the strongest positive correlation was observed between physical activity and exercise adherence, which is highly consistent with the perspectives of self-determination theory and social cognitive theory (19, 23). In the Chinese cultural context, older adult individuals often engage in physical activity with a strong purpose and regularity (56). Once an exercise habit is established, adherence to it tends to be closely linked to daily activity levels (57). Additionally, the negative correlation between exercise adherence and two types of rumination supports the “exercise as a distraction” cognitive mechanism hypothesis (58). Regular exercise may help older adult individuals break the vicious cycle of rumination by redirecting their attention away from negative thoughts (59). By T2, although the correlation between physical activity and exercise adherence remained significant, its strength notably weakened, while the negative correlation between exercise adherence and loneliness became more prominent (60). This change may be closely related to the life circumstances of older adult individuals in China (61). Over time, older adult individuals who consistently exercised might have gained more opportunities for social interaction through continued participation in group sports or community exercise programs, thereby accruing greater benefits in alleviating loneliness (62).

In contrast, the contribution of physical activity alone to psychological benefits may have reached a saturation point (63). Meanwhile, the sense of social integration and belonging fostered by long-term adherence to exercise increasingly plays a crucial role in reducing loneliness (64). This finding provides an important temporal perspective on the role of exercise behaviour in the social and psychological health of older adult individuals in China.

### 4.2 Evolution of cross-sectional network centrality indices

This study examines dynamic changes in the centrality indices of a cross-sectional network. Given that some centrality indices (e.g., closeness) showed only borderline stability at T1, centrality rankings should be interpreted as indicative rather than definitive; accordingly, we focus primarily on expected influence, which demonstrated good stability (65). At T1, stress showed the highest centrality, consistent with psychological responses among older adult individuals in China to multiple challenges associated with ageing, such as physical health decline and changes in social roles (2). According to stress theory (66), stress, as a direct response to real threats, tends to occupy a central position in the early stages of the system (67). It can radiate its influence on other adverse psychological states and health behaviours through mechanisms such as emotional contagion and cognitive appraisal, forming a network structure centred on stress (68). However, by T2, anxiety replaced stress as the most central node in the network, marking a significant theoretical and practical shift. From a developmental psychology perspective, when initial stressors are not effectively alleviated (69), individuals may shift their attention from current stressors to concerns about future uncertainties, thereby transforming stress into anxiety (70). In the context of rapid social changes in China, older adult individuals, facing uncertainties related to healthcare, ageing, and other future challenges, are more likely to develop persistent worry about unknown risks (71). This shift in emotional focus reflects the dynamic characteristics of older adult psychological adaptation—moving from coping with present stressors to worrying about future threats (72). This transition provides crucial temporal insight for stage-specific and targeted psychological interventions.

### 4.3 Temporal relationships in the cross-lagged network

The cross-lagged network analysis in this study revealed the complex longitudinal predictive relationships between physical activity, exercise adherence, and adverse psychological states. First, the significant positive association between exercise adherence and subsequent physical activity aligns closely with the self-efficacy perspective (73). This finding is consistent with results from a longitudinal study, suggesting that individuals who successfully maintain exercise routines develop strong exercise self-efficacy (74). This confidence further encourages them to sustain higher levels of physical activity. In the context of community-based older adult care in China, older adult individuals who consistently participate in organized exercise activities develop stable behavioural patterns, providing important empirical evidence for understanding the mechanisms that sustain health behaviours (75).

Secondly, the observed negative temporal predictive associations between exercise adherence and various adverse psychological states—particularly the prospective links with stress, anxiety, and loneliness—are consistent with the possibility that higher exercise adherence temporally precedes lower subsequent distress (76). This finding is corroborated by a study conducted within the older adult population in China, which suggests that regular exercise not only may be associated with lower stress responses through physiological pathways but, more importantly, in the context of China’s unique collectivist culture, group exercise activities provide older adult individuals with valuable social interaction opportunities, which may be associated with lower loneliness (77, 78). This social support effect is particularly pronounced in community-based exercise programs.

On the other hand, the negative temporal associations between adverse psychological states and health behaviours are also noteworthy. The significant negative association between stress and physical activity is consistent with the basic principles of conservation of resources theory (24). It aligns with a cross-sectional finding that older adult individuals in prolonged stress states tend to reduce physical activity due to the depletion of psychological resources (79). The dual pathway of loneliness, affecting both physical activity and exercise adherence, reflects the unique circumstances of older adult individuals in China, particularly empty nesters (80). The lack of family support not only directly weakens motivation to engage in physical activity but also indirectly affects the maintenance of exercise habits by reducing opportunities for social interaction (81).

Finally, the mutually reinforcing cycle formed within adverse psychological states (stress → depression, stress → anxiety, anxiety → stress) validates the core concepts of emotional network theory (25). It indicates that, in the context of rapid societal changes in China, the multiple sources of stress faced by older adult individuals can create a self-reinforcing network of adverse psychological states through emotional diffusion effects (82). This finding provides an important theoretical basis and practical direction for the development of targeted psychological intervention strategies.

### 4.4 Dynamic significance of cross-lagged network centrality

The centrality analysis of the cross-lagged network reveals asymmetric features in the direction of influence within the variable system. Exercise adherence shows the strongest outwardly expected influence. This finding aligns with the core principles of health behaviour change theories, particularly the approach theory, which emphasizes the dominant role of self-regulation in maintaining health behaviours (83). In empirical research, exercise adherence, as a key indicator of behaviour maintenance, can significantly affect an individual’s emotional state and subsequent activity levels by establishing positive feedback mechanisms (84). In the context of community fitness programs for older people in China, those who consistently engage in exercise often serve as role models within their groups. Their behaviour not only stabilizes their own physical activity levels but also influences their peers’ emotional states through social learning. This may help explain why exercise adherence holds a driving position within the system (85).

On the other hand, physical activity shows the strongest inward expected influence, a pattern consistent with the multi-level interaction perspective of social ecological theory (86). Research suggests that older adult individuals’ daily levels of physical activity are more easily influenced by a combination of personal, interpersonal, and environmental factors (87). In the context of China, external factors such as the accessibility of community facilities, seasonal climate changes, and the level of family support significantly affect older people’s daily activity levels, positioning physical activity in a more passive role within the system (88). Compared to exercise adherence, a behavioural intention indicator, actual physical activity levels are more susceptible to changes in the external environment.

The asymmetric distribution of this centrality structure carries important implications for intervention: Exercise adherence, as a potentially influential node in terms of outward predictive influence, may be considered a priority target of health promotion interventions. Physical activity, as an outcome variable that is more susceptible to influence, requires multi-level environmental support to ensure its stability. This finding provides an important theoretical basis and practical direction for developing targeted health behaviour intervention strategies for older people.

### 4.5 Implications in the era of large language models and human–AI integration

Recent advances in large language models (LLMs) and AI-mediated services may increasingly shape older adults’ information environments, social connectedness, and health-behaviour support. Because our data were collected during 2024–2025, a period of rapid iteration and diffusion of LLM-based applications, it is possible that the strength and configuration of the predictive links observed among physical activity, exercise adherence, and adverse psychological states may evolve as AI companions, conversational agents, and socially assistive robots become more prevalent (89). On the one hand, such tools may provide personalized health education, motivational feedback, and companionship, potentially enhancing adherence and mitigating loneliness-related distress (90); on the other hand, documented cultural tendencies, stereotypes, and other biases in human–LLM interactions may differentially influence users’ psychological experiences, particularly among older adults with varying digital literacy and access (91). Accordingly, our findings should be interpreted within the current technological context, and future research should explicitly assess AI exposure and usage patterns and re-evaluate whether these network patterns remain robust in a future society characterized by deeper human–AI integration.

## 5 Conclusion

This study, using cross-lagged network analysis, systematically reveals the complex dynamic interplay among physical activity, exercise adherence, and adverse psychological states among older adult individuals in China. The findings indicate that exercise adherence emerged as a key node with strong outward predictive influence within the system. It was positively associated with subsequent levels of physical activity and was prospectively associated with lower subsequent levels of several adverse psychological states. In contrast, physical activity is more susceptible to the influence of adverse psychological states and environmental factors. Additionally, the mutually reinforcing network of adverse psychological states and the evolution of centrality from stress to anxiety further emphasize the complexity and dynamic nature of emotional issues in older people. These findings not only provide a new theoretical perspective on the interaction between health behaviours and psychological states in older adult individuals but also offer empirical evidence for developing targeted health promotion strategies. They suggest that future interventions particularly focus on fostering exercise adherence and on addressing dynamic changes in adverse psychological states through multi-level support. Given the regional, predominantly urban sampling, the findings should be generalized cautiously; future studies should replicate these patterns in rural settings and other regions of China.

## 6 Contributions

### 6.1 Theoretical significance

This study introduces the advanced method of cross-lagged network analysis, offering a new theoretical perspective on the relationship between health behaviours and psychological health among older adult individuals. Compared to traditional variable-centred models, the network analysis method overcomes the limitations of linear causal models, revealing a complex dynamic system of interactions among physical activity, exercise adherence, and various adverse psychological states. The study found that exercise adherence occupies a core driving position within the system, deepening understanding of the mechanisms underlying behaviour maintenance in health behaviour theories. Specifically, it provides empirical support for the applicability of self-regulation theory in the older adult population in China. Additionally, the study observed the dynamic evolution of the negative emotion network structure over time, shifting from a stress-centred model to one focused on anxiety. This provides significant longitudinal evidence for the development of emotional network theory. These findings not only enrich the theoretical framework for health promotion in older people but also lay the methodological foundation for future research exploring the complex relationships between health behaviours and psychological states from a dynamic systems perspective.

### 6.2 Practical significance

The findings of this study have important practical implications for developing health promotion strategies for older adult individuals in China. The study highlights the key driving role of exercise adherence within the system, suggesting that practice should place greater emphasis on establishing and maintaining exercise habits, rather than solely focusing on immediate levels of physical activity. Given the dynamic nature of the negative emotion network, interventions should be adjusted according to the characteristics of different stages: in the initial stage, the focus should be on stress management, while in the later stage, attention should shift to anxiety relief. The study also shows that group exercise activities, by providing social support, have unique value in alleviating loneliness among older people. This provides specific direction for the design of community health services. There should be robust development of community-based group exercise programs that not only promote physical health but also meet the social interaction needs of older adult individuals. Moreover, the key predictive pathways identified in the study provide targets for precise interventions, helping to design more targeted and timely health promotion programs that more effectively enhance the physical and mental health of older adult individuals in China.

## 7 Limitations and future directions

This study has several limitations that should be addressed in future research. First, although a longitudinal design was used, data collection at only two time points may limit the ability to capture more complex dynamic processes. Specifically, because we collected only two waves (T1 and T2) spaced 1 year apart, this design cannot capture short-term (daily/weekly) fluctuations or potentially non-linear trajectories, and it limits our ability to probe more complex reciprocal dynamics across multiple intermediate time steps. Moreover, the one-year lag may be too long to detect transient emotion-exercise couplings yet too coarse to identify the most proximal temporal ordering; therefore, the cross-lagged edges should be interpreted as between-wave prospective associations at this particular lag rather than evidence of reciprocal causality. Second, although the sample was drawn from multiple cities, it was concentrated in specific regions, which may limit the generalizability of the findings to the national level. Specifically, participants were recruited from selected cities in Guangdong, Guizhou, and Jiangxi provinces and may not represent older adults living in rural areas, northern China, or other socio-cultural contexts. Accordingly, our conclusions should be interpreted cautiously and should not be overgeneralized to the broader national older-adult population; replication using more geographically and socio-culturally diverse (ideally nationally representative) samples is warranted. Third, although we compared completers and dropouts on observed baseline characteristics and used multiple imputation, attrition may still be associated with unmeasured factors (e.g., health deterioration, functional limitations, or motivation), and multiple imputation relies on the missing-at-random assumption; therefore, some residual attrition bias cannot be ruled out. Future research could strengthen robustness by combining multiple imputation with inverse probability weighting and by conducting sensitivity analyses under missing-not-at-random scenarios. Fourth, all constructs were measured via self-report, which may be susceptible to recall bias, social desirability, and common method variance. Although Harman’s single-factor test suggested that a single factor did not dominate the variance, this approach has been criticised as insufficient for detecting common method bias; therefore, shared method effects may have influenced some within-wave network relationships, particularly for socially sensitive behaviours such as exercise adherence. Future studies should incorporate objective or multi-source measures (e.g., accelerometer-based physical activity, attendance/record-based adherence indicators), and, where feasible, include a marker variable or social desirability measure to enable more robust statistical control (e.g., marker-variable adjustment or latent method factor approaches) and sensitivity analyses. While network analysis can reveal complex relationships between variables, in addition, although expected influence demonstrated good stability, some centrality indices (e.g., closeness) were only borderline stable at T1; therefore, node centrality rankings—especially for less stable indices—should be interpreted cautiously and should not be used to make overly strong claims about specific nodes. It is highly dependent on variable selection, which may lead to the omission of other important variables. Finally, cross-lagged network analysis primarily provides predictive evidence and still requires further experimental research to validate the causal relationships between variables.

Based on the findings and limitations of this study, future research can continue to explore multiple directions. It is recommended to adopt a more intensive longitudinal design with more time points to capture the dynamic interactions among variables better. For example, future work could incorporate three or more waves with shorter intervals (e.g., monthly or quarterly follow-ups) or intensive longitudinal approaches such as daily diaries or ecological momentary assessment (EMA), which are better suited to testing non-linear change and capturing within-person emotion–exercise dynamics. Expanding the sampling scope and including a more diverse older adult population will help validate the generalizability of the research findings. Combining objective measurement methods (such as accelerometers and physiological indicators) with subjective reports can further improve data accuracy. Additionally, future studies could consider incorporating more diverse influencing factors, such as social environment and personal resources, to build a more comprehensive explanatory framework. Finally, developing targeted intervention programs based on the key pathways identified through network analysis and validating their effectiveness through randomised controlled trials would significantly advance the translation of research findings into practice, providing more effective support for health promotion among older adult individuals in China.

## Data Availability

The original contributions presented in the study are included in the article/Supplementary material, further inquiries can be directed to the corresponding authors.
