# Correction: Dynamic interactions between physical activity, exercise adherence, and adverse psychological states in Chinese older adults: a cross-lagged network analysis

**DOI:** 10.3389/fpubh.2026.1841497

**Published:** 2026-04-09

**Authors:** Luyao Xiang, Hao Gou, Jing Yang, Chang Hu, Dong Zhu, Bo Xu

**Affiliations:** 1 Zhuhai Campus, Zunyi Medical University, Zhuhai, China; 2 Hunan University of Medicine, Huaihua, China; 3 College of Physical Education, Qiannan Normal University for Nationalities, Duyun, China; 4 Mianyang Normal University, Mianyang, China; 5 College of Physical Education, Jiangxi Normal University, Nanchang, China

**Keywords:** adverse psychological states, cross-lagged network analysis, dynamic relationship, older adult population in China, exercise adherence, physical activity

In the published article, there was an error in the author affiliations and in the numbering of affiliations in the author line.

The author list and affiliations appeared as:

Luyao Xiang^1†^, Hao Gou^2†^, Jing Yang^3^, Chang Hu^4*^, Dong Zhu^5^ and Bo Xu^6*^

1 Zunyi Medical University, Zhuhai Campus, Zhuhai, China

2 College of Physical Education, Qiannan Normal University for Nationalities, Duyun, China

3 Mianyang Normal University, Mianyang, China

4 College of Physical Education, Jiangxi Normal University, Nanchang, China

5 College of Rehabilitation Medicine and Health, Hunan University of Medicine, Huaihua, China

6 Medical Humanity and Management College, Hunan University of Medicine, Huaihua, China

The correct author list and affiliations appear below:

Luyao Xiang^1,2†^, Hao Gou^3†^, Jing Yang^4^, Chang Hu^5*^, Dong Zhu^2^ and Bo Xu^2*^

1 Zhuhai Campus, Zunyi Medical University, Zhuhai, China

2 Hunan University of Medicine, Huaihua, China

3 College of Physical Education, Qiannan Normal University for Nationalities, Duyun, China

4 Mianyang Normal University, Mianyang, China

5 College of Physical Education, Jiangxi Normal University, Nanchang, China

The original version of this article has been updated.

