# Supplementary material for: Dynamic interactions between physical activity, exercise adherence, and adverse psychological states in Chinese older adults: a cross-lagged network analysis
Source: Front Public Health. 2026 Mar 26;14:1744350. doi: 10.3389/fpubh.2026.1744350 (PMC13062193; doi:10.3389/fpubh.2026.1744350)
Supplement: Supplementary file 1 [file Table_1.docx]

Supplementary Material

# Supplementary Figures and Tables

## Supplementary Tables

**Table S-1** Confirmatory Factor Analysis Model Fit Indices (T1 and T2)

|  | ^2^/df | RMSEA | SRMR | CFI | TLI |
| --- | --- | --- | --- | --- | --- |
| T1- Exercise Adherence | 1.618 | 0.027 | 0.015 | 0.994 | 0.993 |
| T2- Exercise Adherence | 1.560 | 0.025 | 0.016 | 0.994 | 0.993 |
| T1-Depression | 2.323 | 0.039 | 0.014 | 0.995 | 0.992 |
| T2-Depression | 1.890 | 0.032 | 0.013 | 0.996 | 0.993 |
| T1-Anxiety | 1.235 | 0.016 | 0.009 | 0.999 | 0.999 |
| T2-Anxiety | 1.677 | 0.028 | 0.012 | 0.997 | 0.996 |
| T1-Stress | 1.798 | 0.030 | 0.013 | 0.996 | 0.994 |
| T2-Stress | 1.382 | 0.021 | 0.012 | 0.998 | 0.997 |
| T1-Loneliness | 2.178 | 0.037 | 0.013 | 0.995 | 0.992 |
| T2-Loneliness | 1.342 | 0.020 | 0.010 | 0.999 | 0.998 |
| T1-Anger Rumination | 2.835 | 0.046 | 0.019 | 0.985 | 0.982 |
| T2-Anger Rumination | 2.287 | 0.038 | 0.017 | 0.990 | 0.987 |
| T1-Sadness Rumination | 2.092 | 0.035 | 0.016 | 0.991 | 0.989 |
| T2-Sadness Rumination | 3.253 | 0.051 | 0.020 | 0.982 | 0.78 |

**Table S-2** Internal Consistency Reliability Coefficients (Cronbach's α) for Each Study Variable

| Dimension | T1 Cronbach's α | T2 Cronbach's α |
| --- | --- | --- |
| Behavioral Habits | 0.886 | 0.864 |
| Effort Investment | 0.918 | 0.903 |
| Emotional Experience | 0.922 | 0.908 |
| Depression | 0.909 | 0.892 |
| Anxiety | 0.916 | 0.905 |
| Stress | 0.895 | 0.877 |
| Anger Rumination | 0.932 | 0.931 |
| Sadness Rumination | 0.934 | 0.932 |

**Table S-3** Descriptive Statistics and Correlation Matrix for Each Variable at T1 and T2

|  | M±SD | T1PA | T1-EA | T1-DEP | T1-ANX | T1-STR | T1-LON | T1-AR | T1-SR | T2-PA | T2-EA | T2-DEP | T2-ANX | T2-STR | T2-LON | T2-AR | T2-SR |
| --- | --- | --- | --- | --- | --- | --- | --- | --- | --- | --- | --- | --- | --- | --- | --- | --- | --- |
| T1PA | 32.22±32.17 | 1 | .506** | -.199** | -.177** | -.192** | -.211** | -.165** | -.132** | .285** | .157** | -.165** | -.118** | -.123** | -.081* | -0.055 | -0.044 |
| T1-EA | 3.05±0.9 | .506** | 1 | -.293** | -.292** | -.258** | -.294** | -.253** | -.219** | .258** | .269** | -.151** | -.162** | -.198** | -.155** | -.120** | -.076* |
| T1-DEP | 1.72±0.84 | -.199** | -.293** | 1 | .378** | .380** | .276** | .143** | .089** | -.170** | -.135** | .125** | .140** | .104** | .090** | .079* | .123** |
| T1-ANX | 1.65±0.9 | -.177** | -.292** | .378** | 1 | .338** | .286** | .144** | .149** | -.186** | -.142** | .110** | .139** | .154** | .068* | .083* | .133** |
| T1-STR | 1.6±0.87 | -.192** | -.258** | .380** | .338** | 1 | .266** | .126** | .131** | -.203** | -.134** | .159** | .162** | .141** | .084* | .089** | 0.046 |
| T1-LON | 2.6±0.88 | -.211** | -.294** | .276** | .286** | .266** | 1 | .151** | .136** | -.167** | -.146** | .122** | .087* | .137** | .076* | .086* | 0.041 |
| T1-AR | 3.13±1.08 | -.165** | -.253** | .143** | .144** | .126** | .151** | 1 | .224** | -.068* | -0.02 | 0.045 | .077* | 0.023 | -0.013 | 0.052 | .093** |
| T1-SR | 3.02±1.09 | -.132** | -.219** | .089** | .149** | .131** | .136** | .224** | 1 | -.101** | -.089** | .076* | 0.024 | .085* | 0.007 | .109** | .086* |
| T2-PA | 30.81±32.28 | .285** | .258** | -.170** | -.186** | -.203** | -.167** | -.068* | -.101** | 1 | .356** | -.124** | -.077* | -.116** | -.131** | -.120** | -.118** |
| T2-EA | 3.03±0.94 | .157** | .269** | -.135** | -.142** | -.134** | -.146** | -0.02 | -.089** | .356** | 1 | -.235** | -.203** | -.190** | -.281** | -.256** | -.240** |
| T2-DEP | 1.61±0.83 | -.165** | -.151** | .125** | .110** | .159** | .122** | 0.045 | .076* | -.124** | -.235** | 1 | .450** | .431** | .218** | .127** | 0.061 |
| T2-ANX | 1.56±0.89 | -.118** | -.162** | .140** | .139** | .162** | .087* | .077* | 0.024 | -.077* | -.203** | .450** | 1 | .389** | .235** | .116** | .087* |
| T2-STR | 1.51±0.85 | -.123** | -.198** | .104** | .154** | .141** | .137** | 0.023 | .085* | -.116** | -.190** | .431** | .389** | 1 | .192** | .117** | .101** |
| T2-LON | 2.56±0.88 | -.081* | -.155** | .090** | .068* | .084* | .076* | -0.013 | 0.007 | -.131** | -.281** | .218** | .235** | .192** | 1 | .147** | .153** |
| T2-AR | 3.12±1.1 | -0.055 | -.120** | .079* | .083* | .089** | .086* | 0.052 | .109** | -.120** | -.256** | .127** | .116** | .117** | .147** | 1 | .365** |
| T2-SR | 3.05±1.1 | -0.044 | -.076* | .123** | .133** | 0.046 | 0.041 | .093** | .086* | -.118** | -.240** | 0.061 | .087* | .101** | .153** | .365** | 1 |

**Table S-4** T1 Cross-sectional Network Edge Weights (Correlation Coefficients)

| Rank | Node |  | Node | Correlation Coefficient |
| --- | --- | --- | --- | --- |
| 1 | T1-PA | - | T1-EA | 0.447 |
| 2 | T1-DEP | - | T1-STR | 0.224 |
| 3 | T1-DEP | - | T1-ANX | 0.191 |
| 4 | T1-ANX | - | T1-STR | 0.158 |
| 5 | T1-EA | - | T1-AR | -0.143 |
| 6 | T1-EA | - | T1-SR | -0.136 |
| 7 | T1-ANX | - | T1-LON | 0.132 |
| 8 | T1-AR | - | T1-SR | 0.126 |
| 9 | T1-EA | - | T1-DEP | -0.126 |
| 10 | T1-STR | - | T1-LON | 0.120 |
| 11 | T1-EA | - | T1-LON | -0.106 |
| 12 | T1-EA | - | T1-ANX | -0.099 |
| 13 | T1-DEP | - | T1-LON | 0.080 |
| 14 | T1-EA | - | T1-STR | -0.074 |
| 15 | T1-PA | - | T1-LON | -0.055 |
| 16 | T1-LON | - | T1-AR | 0.052 |
| 17 | T1-ANX | - | T1-AR | 0.052 |
| 18 | T1-STR | - | T1-SR | 0.041 |
| 19 | T1-ANX | - | T1-SR | 0.036 |
| 20 | T1-LON | - | T1-SR | 0.032 |
| 21 | T1-DEP | - | T1-AR | 0.026 |
| 22 | T1-PA | - | T1-AR | -0.026 |
| 23 | T1-STR | - | T1-AR | 0.019 |
| 24 | T1-PA | - | T1-STR | -0.015 |
| 25 | T1-PA | - | T1-DEP | -0.006 |
| 26 | T1-PA | - | T1-ANX | -0.004 |

**Table S-5** T2 Cross-sectional Network Edge Weights (Correlation Coefficients)

| Rank | Node |  | Node | Correlation Coefficient |
| --- | --- | --- | --- | --- |
| 1 | T2-PA | - | T2-EA | 0.269 |
| 2 | T2-DEP | - | T2-ANX | 0.258 |
| 3 | T2-DEP | - | T2-STR | 0.253 |
| 4 | T2-AR | - | T2-SR | 0.224 |
| 5 | T2-ANX | - | T2-STR | 0.192 |
| 6 | T2-EA | - | T2-LON | -0.156 |
| 7 | T2-EA | - | T2-AR | -0.146 |
| 8 | T2-EA | - | T2-SR | -0.125 |
| 9 | T2-EA | - | T2-DEP | -0.098 |
| 10 | T2-ANX | - | T2-LON | 0.089 |
| 11 | T2-DEP | - | T2-LON | 0.048 |
| 12 | T2-STR | - | T2-LON | 0.045 |
| 13 | T2-EA | - | T2-STR | -0.042 |
| 14 | T2-LON | - | T2-SR | 0.036 |
| 15 | T2-EA | - | T2-ANX | -0.033 |
| 16 | T2-LON | - | T2-AR | 0.027 |
| 17 | T2-ANX | - | T2-AR | 0.023 |
| 18 | T2-STR | - | T2-AR | 0.008 |

**Table S-6** T1 and T2 Cross-sectional Network Node Expected Influence (EI)

| Rank | Node | Expected Influence |
| --- | --- | --- |
| 1 | T1-PA | 0.439 |
| 2 | T1-EA | -1.972 |
| 3 | T1-DEP | 0.636 |
| 4 | T1-ANX | 0.951 |
| 5 | T1-STR | 0.989 |
| 6 | T1-LON | 0.075 |
| 7 | T1-AR | -0.543 |
| 8 | T1-SR | -0.575 |
| 9 | T2-PA | 0.185 |
| 10 | T2-EA | -1.964 |
| 11 | T2-DEP | 0.871 |
| 12 | T2-ANX | 1.112 |
| 13 | T2-STR | 0.851 |
| 14 | T2-LON | -0.460 |
| 15 | T2-AR | -0.297 |
| 16 | T2-SR | -0.297 |

**Table S-7** Regression Path Coefficients in the Cross-lagged Network (Autoregressive and Cross-lagged Paths)

| Rank | Node | → | Node | Regression Type | Regression Coefficient |
| --- | --- | --- | --- | --- | --- |
| 1 | EA | → | EA | Autoregressive | 0.233 |
| 2 | PA | → | PA | Autoregressive | 0.210 |
| 3 | EA | → | PA | Cross-Lagged | 0.131 |
| 4 | EA | → | STR | Cross-Lagged | -0.129 |
| 5 | STR | → | PA | Cross-Lagged | -0.108 |
| 6 | ANX | → | SR | Cross-Lagged | 0.084 |
| 7 | STR | → | DEP | Cross-Lagged | 0.081 |
| 8 | EA | → | ANX | Cross-Lagged | -0.081 |
| 9 | EA | → | LON | Cross-Lagged | -0.079 |
| 10 | STR | → | ANX | Cross-Lagged | 0.078 |
| 11 | DEP | → | SR | Cross-Lagged | 0.074 |
| 12 | ANX | → | STR | Cross-Lagged | 0.070 |
| 13 | EA | → | AR | Cross-Lagged | -0.065 |
| 14 | PA | → | DEP | Cross-Lagged | -0.056 |
| 15 | LON | → | PA | Cross-Lagged | -0.055 |
| 16 | ANX | → | PA | Cross-Lagged | -0.055 |
| 17 | STR | → | STR | Autoregressive | 0.054 |
| 18 | LON | → | STR | Cross-Lagged | 0.054 |
| 19 | LON | → | EA | Cross-Lagged | -0.050 |
| 20 | DEP | → | PA | Cross-Lagged | -0.048 |
| 21 | AR | → | EA | Cross-Lagged | 0.046 |
| 22 | SR | → | AR | Cross-Lagged | 0.045 |
| 23 | ANX | → | EA | Cross-Lagged | -0.038 |
| 24 | AR | → | SR | Cross-Lagged | 0.036 |
| 25 | ANX | → | ANX | Autoregressive | 0.035 |
| 26 | DEP | → | ANX | Cross-Lagged | 0.035 |
| 27 | STR | → | EA | Cross-Lagged | -0.035 |
| 28 | EA | → | DEP | Cross-Lagged | -0.033 |
| 29 | AR | → | STR | Cross-Lagged | -0.031 |
| 30 | SR | → | SR | Autoregressive | 0.031 |
| 31 | DEP | → | EA | Cross-Lagged | -0.029 |
| 32 | LON | → | DEP | Cross-Lagged | 0.029 |
| 33 | DEP | → | DEP | Autoregressive | 0.026 |
| 34 | SR | → | STR | Cross-Lagged | 0.024 |
| 35 | STR | → | AR | Cross-Lagged | 0.022 |
| 36 | SR | → | EA | Cross-Lagged | -0.019 |
| 37 | SR | → | PA | Cross-Lagged | -0.015 |
| 38 | LON | → | AR | Cross-Lagged | 0.014 |
| 39 | SR | → | DEP | Cross-Lagged | 0.009 |
| 40 | ANX | → | DEP | Cross-Lagged | 0.009 |
| 41 | ANX | → | AR | Cross-Lagged | 0.005 |
| 42 | DEP | → | AR | Cross-Lagged | 0.001 |

**Table S-8** Inward and Outward Expected Influence (InStrength / OutStrength) in the Cross-lagged Network

| node | InStrength | node | OutStrength |
| --- | --- | --- | --- |
| ANX | 0.193 | ANX | 0.261 |
| AR | 0.152 | AR | 0.112 |
| DEP | 0.216 | DEP | 0.187 |
| EA | 0.217 | EA | 0.518 |
| LON | 0.079 | LON | 0.202 |
| PA | 0.412 | PA | 0.056 |
| SR | 0.193 | SR | 0.113 |
| STR | 0.308 | STR | 0.323 |

## Supplementary Figures


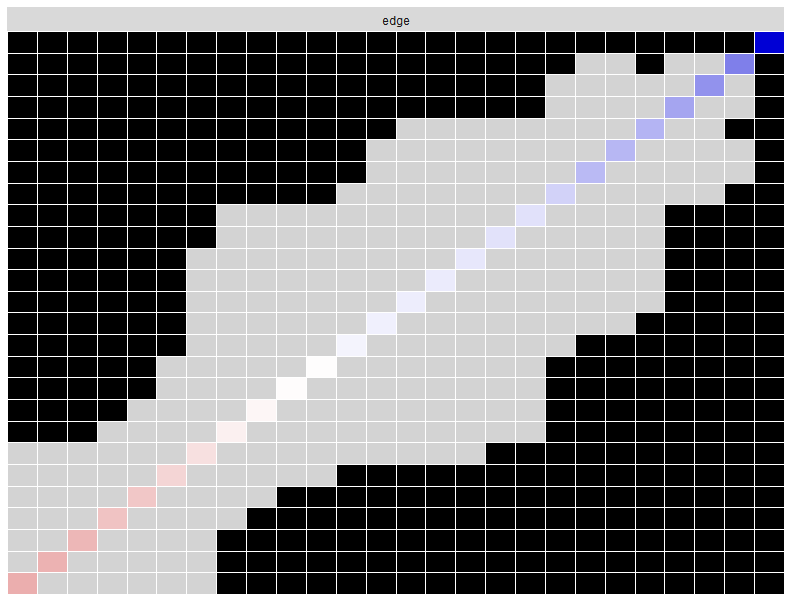


**Figure S-1** T1 Cross-sectional Network Edge Weights Bootstrap Confidence Intervals

Note: This figure displays the Bootstrap confidence intervals for the edge weights in the T1 cross-sectional network. The confidence intervals help assess the stability and reliability of the network estimates.


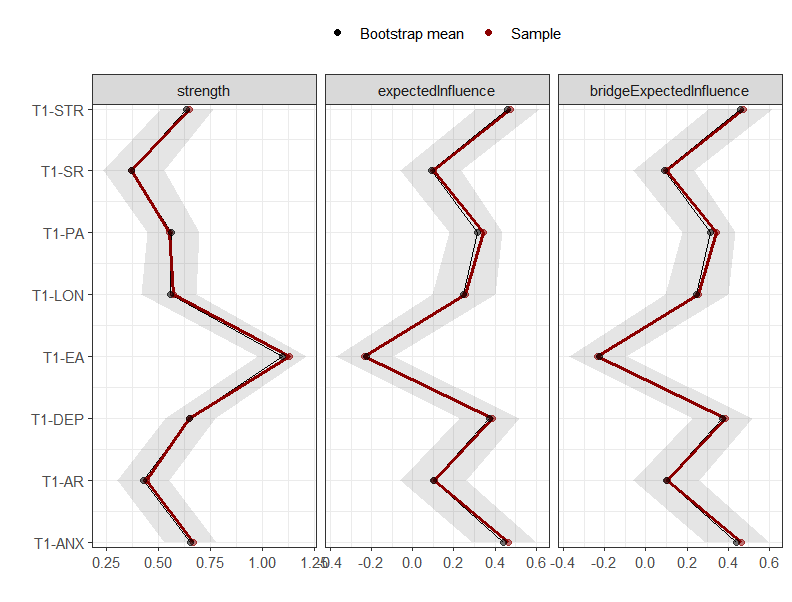


**Figure S-2 Stability Test of T1 Cross-sectional Network Centrality Indices**

Note: This figure would typically illustrate the results of the stability test for the centrality indices of the T1 cross-sectional network, showing how stable the centrality measures (e.g., Expected Influence, InStrength, OutStrength) are for each node across bootstrap iterations or other methods of assessing reliability.


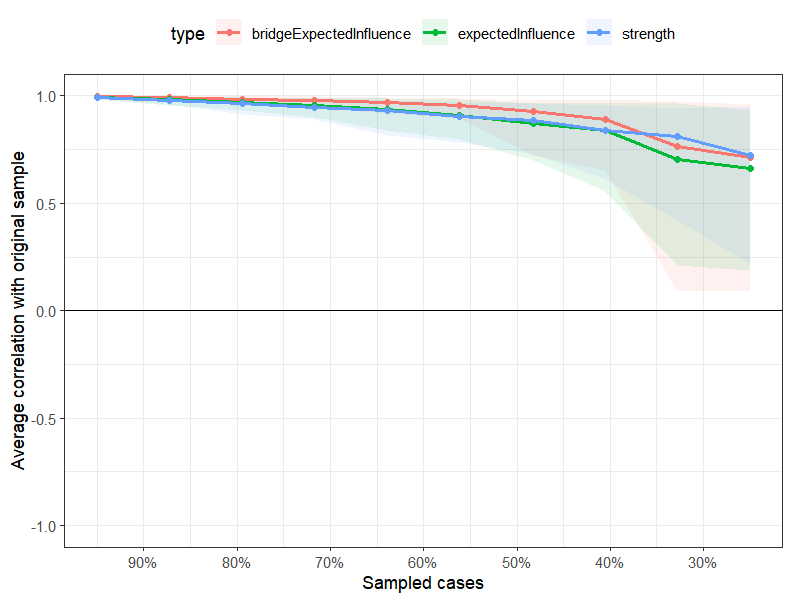


**Figure S-3** Bootstrap Test of Node Centrality Differences in T1 Cross-sectional Network


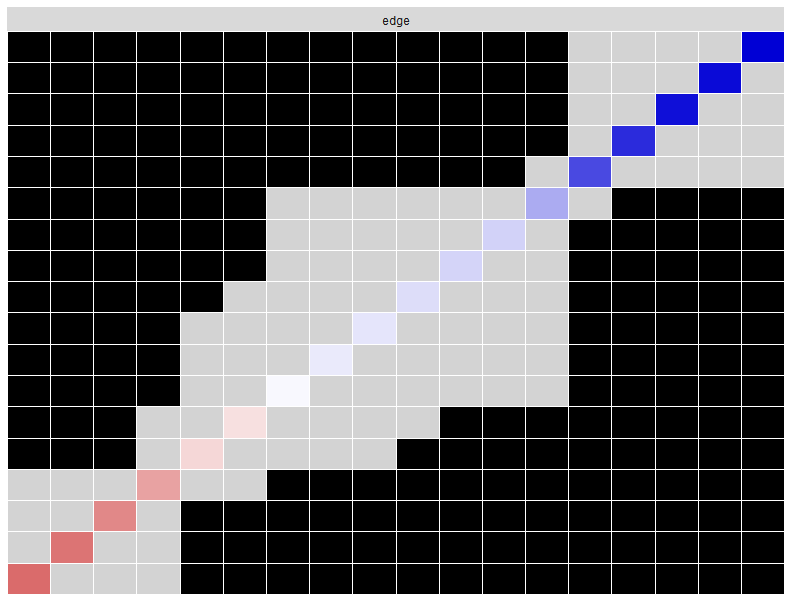


**Figure S-4** Bootstrap Confidence Intervals for Edge Weights in T2 Cross-sectional Network


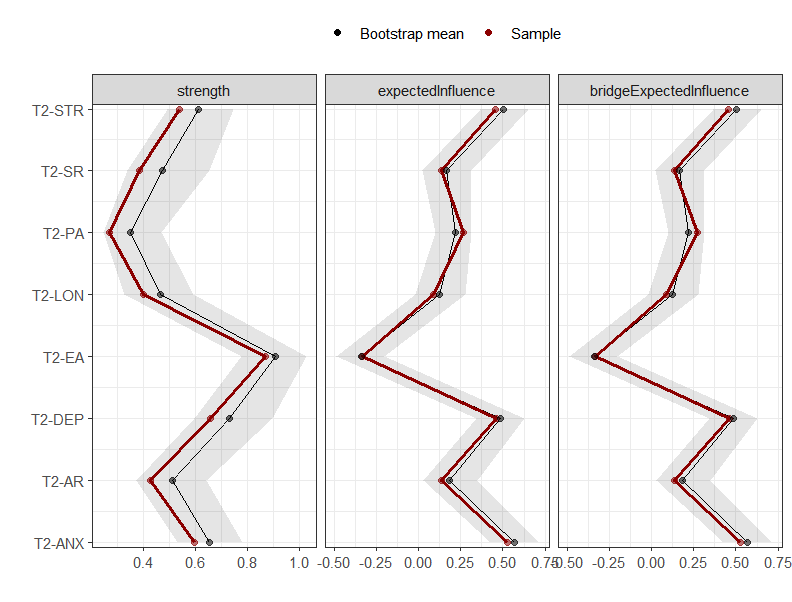


**Figure S-5** Stability Test of T2 Cross-sectional Network Centrality Indices

****
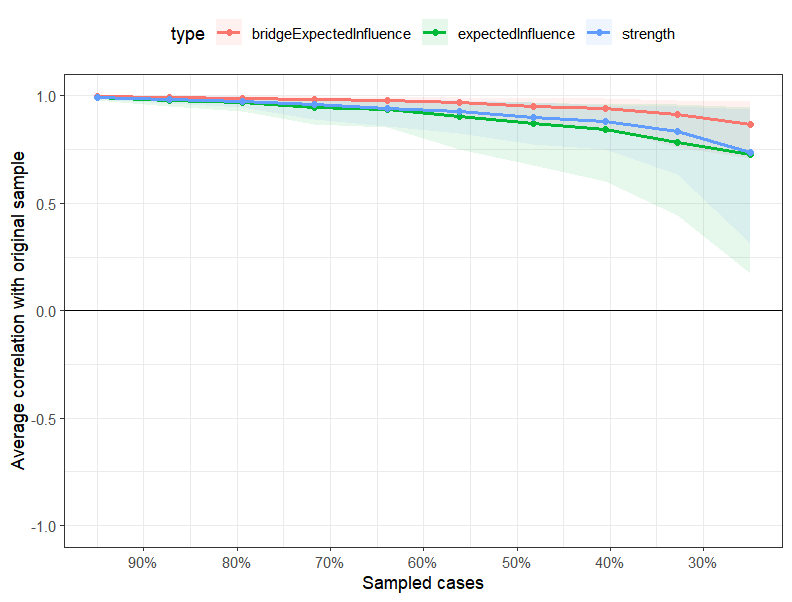
****

**Figure S-6** Bootstrap Test of Node Centrality Differences in T2 Cross-sectional Network

**
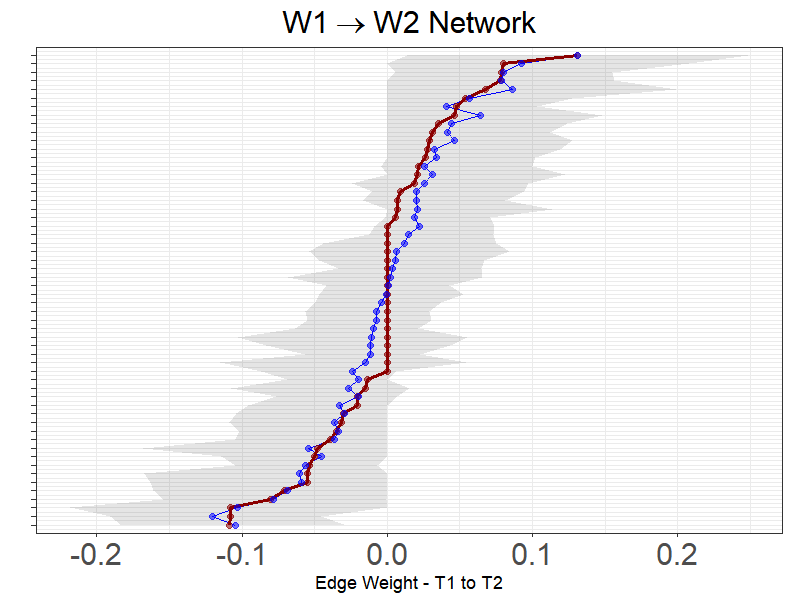
**

**Figure S-7** Bootstrap Confidence Intervals for Edge Weights in the Cross-lagged Network

Note: This figure would typically display the Bootstrap confidence intervals for the edge weights in the cross-lagged network, showing the variability or uncertainty around the estimated correlation coefficients between the variables. The confidence intervals provide insight into the stability of the relationships between variables over time, considering the resampling method used to assess their reliability.
